# WHANAUNGATANGA AND FILIAL PIETY: INTERGENERATIONAL EXCHANGES IN CONTEMPORARY MAORI AND KOREAN CULTURES

**DOI:** 10.1093/geroni/igz038.147

**Published:** 2019-11-08

**Authors:** Hong-Jae Park, Jim Anglem

**Affiliations:** 1 Western Sydney University, Sydney, Australia; 2 University of Canterbury, Christchurch, New Zealand

## Abstract

Every culture has its own tradition of intergenerational exchange based on accepted norms, while the meanings of traditional filial values have evolved over time. This paper aims to identify the various forms of filial care, support and respect for older people in Maori and Korean cultures, and reconceptualise current ways of intergenerational exchanges in both physical and virtual contexts. Data were collected through a qualitative inquiry framework consisting of 32 individual interviews and 5 ethnographic observations in New Zealand and South Korea. Thematic analysis of the data was used to identify themes and patterns from the participants’ perspectives and experiences in the multilingual research context. In this cross-cultural study, for Māori participants, whanaungatanga (family relationships) was recognised as a core value that places whanau (family) at the centre of whakapapa (human and non-human relations). For Korean participants, their tradition of filial piety has continued to constitute a major component of familism mindsets and practices, while their ability to support their parents and maintain connections to their ancestors varied. Being knowledgeable about the traditional values of intergenerational solidarity helped generations feel connected and supported by each other, although both monetary and non-monetary support for one’s elders has come under strain due to the impact of changes in family ties and social dynamics. Technological developments have reshaped traditional filial practices, offering new ways of intergenerational exchanges. Redefining whanaungatanga and filial piety can provide a theoretical basis for developing the concept of extended social work through avoiding excessive individualism and culture-blind approaches.

